# Integrating Community-Based Learning Into the Classroom: Students Become Long-Term Care Resident Advocates

**DOI:** 10.1093/geroni/igaf122.1590

**Published:** 2025-12-31

**Authors:** Katherine Kwong, Claire Benedict

**Affiliations:** Connecticut College, New London, Connecticut, United States; The Holleran Center for Community Action and Public Policy, Connecticut College, New London, Connecticut, United States

## Abstract

Community-based learning (CBL) is a teaching method that develops critical and reflective thinking on social and civic issues by combining academic instruction with community work by partnering with local community groups. The most successful CBL courses are built around a partnership between all participants (community partners, faculty, and students). CBL provides students with practical experiences of their academic studies and an understanding of ethical community engagement. In 2024 KFF notes that there were approximately 1.2 million individuals residing in certified nursing home facilities. In the state of Connecticut, KFF estimates that there are approximately 20,000 residents, with the majority being older adults. The Connecticut Long-Term Care Ombudsman Program (CT-LTCOP) provides an essential service as advocates for individuals who reside in long-term care facilities (LTC) as the eight Regional Ombudsman work to resolve complaints and concerns of residents and offer assistance and support. Their critical work is aided by Volunteer Resident Advocates (VRAs) assigned to select buildings. Elder Abuse was a course offered in partnership with CT-LTCOP with the goal of integrating CBL into the classroom, resulting in students becoming certified Volunteer Resident Advocates at the completion of the course. The main goals for integrating CBL into the classroom were: (1) provide an opportunity for undergraduate students to connect concepts learned in the classroom to their work within the LTC community; and, (2) to develop a model for faculty on building an effective CBL curriculum that benefits the students and the community.

